# Exploring Changes in Parental Feeding Practices With Young Children Over Time and Their Influence on Young Children's Vegetable Consumption

**DOI:** 10.1111/mcn.70238

**Published:** 2026-09-14

**Authors:** Luke Pullar, Alison C. Spence, Megan Jarman, Jacqueline Blissett, Karen J. Campbell, Alissa Burnett

**Affiliations:** ^1^ Institute for Physical Activity and Nutrition Deakin University Geelong Melbourne Australia; ^2^ School of Psychology, Health and Clinical Science Aston University Birmingham UK

**Keywords:** longitudinal, parental feeding practices, vegetable consumption, young children

## Abstract

Parental feeding practices have been associated with young children's vegetable consumption cross‐sectionally, but there is limited longitudinal evidence that measures multiple feeding practices at multiple time points with a robust dietary outcome measure. This longitudinal, prospective study aims to explore whether feeding practices (or change in feeding practices) are associated with vegetable consumption in young children. In 2010–2014, 255 first‐time parents self‐reported feeding practices via the Comprehensive Feeding Practices Questionnaire, and child diets via multiple 24‐h recalls when their children were 1.6, 3.6, and 5 years old. Linear mixed modelling assessed the change in parental feeding practices and associations with child vegetable consumption. Monitoring (β = 0.38), food rewards (β = 0.23), and restriction for health (β = 0.13) all increased as children got older (p < 0.001). Parents who covertly controlled their children's diets at age 1.6 years had children who consumed 11.0 g/day more vegetables 2 years later. Parents who eat more vegetables at child age 1.6 years old had children who consumed 8.8 g/day more vegetables 2 years later and 12.2 g/day more vegetables 3.5 years later. In contrast, parents who reported higher use of emotion regulation at child age 1.6 years had children who consumed 33.7 g/day less vegetables 3.5 years later. Parents who increased their use of covert control and food rewards throughout the study had children consuming 13.6 g/day more and 11.6 grams/day less vegetables, respectively. Therefore, parents should be supported to use more covert control, consume more vegetables themselves and use less emotion regulation and food rewards, to support their children's vegetable consumption.

## Summary

- Parents report greater use of some feeding practices as their children get older, in particular monitoring their children's food consumption, using food as a reward, and restricting their children's diets.
- Parents' use of more structure‐based feeding practices (such as modelling and covert control) is associated with increased vegetable consumption in young children over time.
- Parents' use of more coercive control‐related feeding practices (such as use of food for emotion regulation and food as a reward) is associated with reduced vegetable consumption in young children over time.

## 1 Introduction

Young children's diets, specifically their vegetable intakes, are suboptimal (Aune et al. 2017), with only 20.1% and 2.1% of children aged 2‐ to 3‐ and 4‐ to 8‐years meeting vegetable recommendations in Australia (Australian Bureau of Statistics 2023). Furthermore, vegetables are the food group consumed least in line with dietary guidelines internationally (Micha et al. 2015). This is of particular concern as vegetables are a major source of vitamins, minerals, and fibre (World Health Organisation 2020); and are a protective factor against the future development of noncommunicable diseases such as diabetes, heart disease, stroke, and some types of cancer (Aune et al. 2017; Cooper et al. 2012; Jeurnink 2012). In addition, previous research indicates that dietary habits (including consuming enough vegetables) formed in early childhood (1–5 years) are often sustained into adulthood (da Costa et al. 2024; Dubois et al. 2022). Therefore, it is important that young children develop healthy dietary habits, such as meeting dietary guidelines for vegetables, to maximise their health in the future. Thus, understanding ways to increase vegetable consumption early in life is an important focus for research.

Parents are the main gatekeepers to their children's diets and have a strong influence on their children's vegetable consumption (Blissett 2011; Jarman et al. 2022; Vaughn et al. 2016), particularly before their children start school (Savage et al. 2007). Therefore, it is necessary to understand how parents influence their children's diets. An influence of particular interest is the techniques that parents use when feeding their children, known as ‘parental feeding practices’ (Blissett 2011; Vaughn et al. 2016). Previous research suggests that parental feeding practices alone account for approximately 20% of the variance in fruit and vegetable consumption amongst children aged 2–7 years (Taylor et al. 2017). While the remaining 80% of the variance in fruit and vegetable consumption may be explained by multiple influences over multiple ecological levels (Jarman et al. 2022), feeding practices are modifiable behaviours which show particularly strong relationships with vegetable consumption rather than with fruit consumption in prospective studies (Pullar et al. 2025). Thus, given the potential for change in feeding practices, even small changes may yield a valuable improvement in children's vegetable consumption at the population level.

As parental feeding practices can help regulate their children's consumption of vegetables (Blissett 2011; Pullar et al. 2025; Taylor et al. 2017; Vaughn et al. 2016), it is important to understand which feeding practices can have a positive influence, and which could negatively influence their children's vegetable consumption. Parental feeding practices have previously been categorised into three main constructs: coercive control, structure, and autonomy support (Vaughn et al. 2016). Coercive controlling feeding practices relate to the parents' attempts to apply external pressure to impose the parents will upon the child (Vaughn et al. 2016). Structure‐based feeding practices refer to how parents organise their children's eating environments to facilitate desired behaviour (Vaughn et al. 2016). While autonomy supportive feeding practices relate to techniques that parents use when feeding their children that promote the child to become more independent (Vaughn et al. 2016). Previous research indicates that structure‐based feeding practices such as modelling and promoting vegetable availability are particularly strongly associated with better vegetable consumption in young children (Blissett 2011; Pullar et al. 2025; Taylor et al. 2017).

While feeding practices in the autonomy support category, such as encouragement, involving the child in the meal preparation or mealtime itself, and nutrition education, are also thought to be associated with improved vegetable outcomes in young children (De Bock et al. 2012; Pullar et al. 2025; Weinfield et al. 2020; Yee et al. 2017), the findings are not as strong as those of structure‐based feeding practices (such as modelling) in the existing literature. On the other hand, coercive controlling feeding practices (such as pressure to eat and restriction) have been associated with poorer vegetable consumption in young children (Blissett 2011; Pullar et al. 2025; Warkentin et al. 2020; Wood et al. 2020). The outcomes of feeding practices are relatively complex because of the potential for differing influences over time. For example, pressure to eat has been suggested to lead to short‐term increases in consumption of the target food (i.e., vegetables) but can lead to negative outcomes over time (Gregory et al. 2011; Pullar et al. 2025). Little is known about the stability of feeding practices in early childhood (1–5 years) as children get older. To our knowledge only one study has explored longitudinal changes of numerous parental feeding practices with multiple follow ups, which reported small changes over time, particularly when the children were 1–5 years (Eichler et al. 2019), therefore further research is warranted.

The feeding practices that parents use when feeding their children also show associations with several factors such as socioeconomic status, education, marital status, child age, child BMI, child temperament, and child fussiness (Jarman et al. 2022; Ohly et al. 2013; Pearson et al. 2009; Zarnowiecki et al. 2014). Therefore, it could be hypothesised that parental use of different feeding practices would also change over time in response to changes in such child characteristics (Burnett et al. 2022; Russell and Russell 2019; Saltzman et al. 2018). However, most of the existing literature exploring the associations between feeding practices and vegetable consumption utilises a cross‐sectional design, with limited longitudinal data and uses brief FFQ measures of child diet (Blissett 2011; Pullar et al. 2025). This is problematic as longitudinal study design supports the understanding of causal pathways rather than finding simple associations between two variables (Wang et al. 2017). A recent systematic review highlighted the lack of longitudinal studies exploring the associations between feeding practices and fruit and vegetable consumption, with some feeding practices being scarcely investigated and not being specifically assessed at multiple time points (Pullar et al. 2025).

Given the lack of longitudinal research that has assessed feeding practices at multiple time points alongside robust measures of dietary outcomes, and the lack of studies that explore the stability of feeding practices over time, this study had two aims. The first aim was to examine whether parental feeding practices change as children age across early childhood (1.5–5 years). Based on previous research (Eichler et al. 2019), it is expected that small changes in reported feeding practices will occur. The second aim was to investigate whether feeding practices (or the change in feeding practices) impact later vegetable consumption (either in quantity or variety) in young children. In line with previous findings (Pullar et al. 2025; Gubbels et al. 2020), it was expected that structure‐based feeding practices (such as modelling) will have a positive influence on vegetable consumption, and coercive controlling feeding practices (such as pressure) will have a negative influence on vegetable consumption.

## 2 Method

### 2.1 Study Design

Data for this study were from the Melbourne Infant Feeding, Activity and Nutrition Trial (InFANT) Programme. This programme was an obesity prevention cluster‐randomised controlled research trial, with the intervention trial phase conducted between 2008 and 2010. Follow up data collection after intervention cessation then occurred between 2010 and 2014. Infants were aged on average 4 months at baseline of the trial. The full methodology of the Melbourne InFANT Programme is described elsewhere, including the diet, physical activity, and sedentary behaviour intervention (Campbell et al. 2008; Campbell et al. 2013). Briefly, the programme aimed to improve the diet quality of young children through improving the primary caregiver's knowledge of feeding and nutrition, feeding practices, self‐efficacy for promoting healthy eating and modelling. The 15‐month intervention was delivered via quarterly group sessions, which involved a 2‐h session delivered by a dietician. The control group received non‐obesity focused newsletters on other aspects of child development. Relevant data was collected at baseline, post intervention (1.6 years old), and at two further follow up points (child ages approximately 3.6 and 5 years). The present study utilised data on parental feeding practices and child vegetable consumption.

### 2.2 Participants

The Melbourne InFANT Programme recruited first time parents of young infants from 14 local government areas (LGAs) in Victoria, Australia in 2008. First‐time parent groups within LGAs were enlisted (*n* = 62), which resulted in 542 parents being recruited. These groups were predominantly attended by mothers, meaning all the study participants (*n* = 530) at baseline were mothers. If groups declined to participate or did not meet inclusion criteria (minimum of eight within a group consenting to participate, or six in low socioeconomic areas), the next group in the randomly generated list was approached.

### 2.3 Measures

#### 2.3.1 Demographic Data Collection

Self‐reported questionnaires were used to collect demographics at baseline. Parents reported the child's birthdate, child's sex, their highest level of education (trade/diploma or lower/university or higher), their own birthdate, their marital status (living with partner/not living with partner), and self‐reported pre‐pregnancy height and weight (to calculate their BMI). Both education and marital status were dichotomised for this analysis and reporting. Child height and weight were measured (to calculate zBMI) at each time point by trained research staff.

#### 2.3.2 Feeding Practices

The person identifying as the primary caregiver was asked to complete the survey of parent feeding practices at each time point. Ten parental feeding practices were assessed at the three follow up points in the Melbourne InFANT Programme (child aged 1.5, 3.5, and 5 years). Seven of the feeding practices were subscales from the Comprehensive Feeding Practice Questionnaire (CFPQ; Musher‐Eizenman and Holub 2007). These were the use of pressure to eat (e.g., if my child says ‘I am not hungry’, I try to get him/her to eat anyway), offering food rewards (e.g., I offer my child his/her favourite foods in exchange for good behaviour), restriction for health (e.g., I have to be sure that my child does not eat too much of his/her favourite foods), modelling (e.g., I try to eat healthy foods in front of my child, even if they are not my favourite), encouraging balance and variety (e.g., I encourage my child to eat a variety of foods), the use of food for emotion regulation (e.g., do you give your child food/drink if s/he is upset even if you think s/he is not hungry?), and monitoring (e.g., how much do you keep track of the sweets that your child eats?). The subscales included three to four questions each, measured on a 5‐point scale. The scores were averaged to obtain a continuous score for each feeding practice. The CFPQ has been validated in children as young as 18 months and was the best available measure for the age group at the time of the programme.

In addition, two other feeding practices were assessed. These were availability of vegetables and covert control. Availability was a single item measured that asked, ‘how often are vegetables other than potato in your home?’ and was measured on a 4‐point Likert scale, ranging from never to always. Covert control refers to parent controlling the consumption of certain foods without the child knowing (Ogden et al. 2006). The tool consisted of five questions (e.g., do you not buy foods that you want because you do not want your child to have them?), where responses were answered on a 5‐point Likert scale, ranging from never to always (Ogden et al. 2006). The covert control score was calculated as the mean score of the responses, similarly to the CFPQ items.

##### 2.3.2.1 Parent Vegetable Intake

Parental vegetable intake was assessed using a food frequency questionnaire (FFQ), in which one item asked parents how many servings of vegetables they typically eat per day. The responses could range from none to seven servings per day.

#### 2.3.3 Vegetable Consumption

Children's vegetable consumption (both quantity and variety) was assessed via multiple 24‐h recalls, which have been reported in detail elsewhere (Campbell et al. 2008; Campbell et al. 2013; Spence et al. 2013). Diet could not be assessed at baseline (when children were 4 months of age and not yet eating) but was assessed at the follow‐up time points. Parents were given a food measurement booklet to assist in their estimation and completed three unscheduled telephone recalls at each time point. The telephone recalls followed the US Department of Agriculture 5‐step multi‐pass method where participants were asked to report all food and beverages consumed in 24‐h period, then asked a series of questions from nine food category questions for any additional foods they may have forgotten, then asked the time and occasion when they consumed the foods, were asked to provide descriptions and amounts of each food reported and review each eating occasion and between occasions, and lastly a final probe for anything else consumed (Conway et al. 2003). These recalls were conducted by trained, blinded research staff and coding of the recalls were checked by a dietician for accuracy. A disaggregation approach like the method to calculate MyPyramid Food Groups by the United States Department of Agriculture (Bowman et al. 2008) was used to calculate the quantity of vegetables consumed per day (excluding potatoes), then the average grams of vegetables over the recalled 3 days was calculated. Vegetable variety was calculated by summing the number of specific vegetable types reported each day and calculating the average across the three recalls. Mixed foods were designated a vegetable variety score of two or three corresponding to their reported ingredients (Hesketh et al. 2020).

### 2.4 Statistical Analysis

The statistical analyses and data cleaning were conducted on Stata 18 (StataCorp 2023). Only participants with complete data for their feeding practices and dietary outcomes at any time point were included in this analysis. Participants from both intervention and control groups were included in this analysis and treatment arm was controlled for the analysis. If any demographic data were missing, participants were still included in the analysis, as long as no data was missing for their feeding practices and vegetable outcomes for that time point. Data from T2 (child age 9 months) were not included in this analysis as no data on feeding practices were collected. Tests for internal consistency amongst the feeding practice items were conducted for each time point, using Cronbach alpha tests (see Supporting Information). If tests showed below a moderate level of internal consistency (*α* < 0.6), alpha tests were rerun to explore whether removing any items would improve agreement, and one item was removed from the restriction for health subscale. Additionally, multicollinearity checks were done, with the variance influence factors for all feeding practices scoring below 1.25—indicating no issues with collinearity. Descriptive statistics (frequencies and percentages) were used to describe the characteristics of the included sample and the full sample from the Melbourne InFANT Programme so a comparison could be made.

Linear mixed modelling was used to assess whether there were any changes in each feeding practice per 1‐year increase in child age, with each individual feeding practice being a dependent variable, and child age as the independent variable. Child age was used rather than categorical timepoints to account for both the unequal spacing between the timepoints (2 years between T3 and T4, and 1.5 years between T4 and T5) and the variability in children's ages within each wave. This allows model coefficients to be interpreted as the change in feeding practice score per 1‐year increase in child age. This model had three levels with the self‐reported feeding practice score being nested within the individual, and the individual nested within the parent group they were recruited from (to account for potential shared characteristics, such as similar environments or parenting resources). Linear mixed modelling was also used to assess whether any of the reported feeding practices (or changes in feeding practices) were associated with later vegetable consumption, with vegetable consumption being the dependent variable and the reported feeding practice score as the independent variable in the model. Feeding practice scores from when the children were ~1.6 years old were used (as this was the first time point when these were assessed) and both vegetable outcomes (quantity and variety) were used when the children were ~3.6 years and ~5 years old. This model was adjusted for reported vegetable consumption when the children were ~1.6 years (as a covariate) to assess the change in vegetable consumption as the children got older. Given the known importance of vegetable intakes and feeding practices before 1.5 years of age, it is acknowledged this is a conservative approach which could underestimate effects, and therefore a sensitivity analysis was also conducted without the covariate of child vegetable consumption at 1.6 years (see Supporting Information). For the feeding practices that significantly changed over time, additional linear mixed models were used to assess whether these changes in the feeding practices were concurrently associated with any changes in vegetable outcomes. Separate models were used for each feeding practice as the independent variable and each vegetable outcome at each time point as the dependent variable. All the above regression models were adjusted for time‐fixed (treatment arm, child sex, maternal education, parent age, child age) and time‐varying (relation to child, marital status, parent BMI, and child zBMI) characteristics.

Post hoc power analyses were done to determine the power achieved for each analysis. For the first research question, the effect size in the linear mixed model was calculated (effect size = 0.049). Using *α* = 0.05 for two‐tailed tests and 10 predictors in the model, the power achieved for this model was 0.94 (a sample size of 163 would have been sufficient to achieve 0.8 power). For the second research question, the effect size in this model was calculated (effect size = 0.09). Again, using *α* = 0.05 for two‐tailed tests and 11 predictors, the power achieved in this model was 0.99 (a sample size of 86 would have been sufficient to achieve 0.8 power).

### 2.5 Ethics Statement

Ethics approval for the initial trial and the follow‐up (as an extension) was received from Deakin University's Human Research Ethics Committee (EC 175–2007) and the Victorian Government Department of Human Services, Office for Children, Research Coordinating Committee. Written informed consent was required from participants to take part in the initial trial, and participants were required to complete a new written consent form to take part in the follow‐up assessments.

## 3 Results

Of the 530 participants who commenced the Melbourne InFANT programme, 255 (47.0%) parents were retained until the 5‐year‐old follow‐up (T5), with 254 participants providing complete data at all time points. Table 1 shows the descriptive characteristics of the included participants at baseline, and at each follow up time point. Just over half of the children were male (52.9%), with a mean age of 0.3 years at baseline (T1), 1.6 years at time point 3 (T3), 3.6 years at time point 4 (T4), and 5.0 years at time point 5 (T5). All parents were the child's mother at T1, with a mean age of 32.8 years at T1. Nearly all parents were living with a partner (98.8%), and over half were educated at least to university level (60.8%). There were no differences between the demographics of the sample included in this analysis and the full sample from the InFANT programme, therefore any missing participants were assumed to be missing completely at random. See Supporting Information for a table of the descriptive characteristics for the included sample and full sample.

**Table 1:** Sample descriptive characteristics included in the regression model.

		T1	T3	T4	T5
Parent	Age (years)	32.8 (4.1)			
	BMI	24.0 (4.7)	25.6 (5.4)	25.6 (5.5)	25.6 (5.6)
	Relation to child				
	Mother	255 (100.0%)	249 (99.6%)	254 (99.6%)	254 (99.6%)
	Father	0	1 (0.4%)	1 (0.4%)	1 (0.4%)
	Education				
	Trade/Diploma or lower	100 (39.2%)			
	University or higher	155 (60.8%)			
	Living with partner				
	Yes	252 (98.8%)	241 (98.8%)	241 (94.9%)	242 (94.9%)
	No	3 (1.2%)	3 (1.2%)	13 (5.1%)	13 (5.1%)
Child	Age (years)	0.3 (0.1)	1.6 (0.2)	3.6 (0.2)	5.0 (0.1)
	zBMI	−0.46 (1.04)	0.83 (0.99)	0.63 (0.87)	0.53 (0.97)
	*Sex*				
	Girl	120 (47.1%)			
	Boy	135 (52.9%)			
Group	Intervention	129 (50.6%)			
	Control	126 (49.4%)			

Children's vegetable consumption increased from a mean of 85.5 grams per day when the children were 1.6 years to 91.6 grams per day and 122.3 grams per day on average when the children were 3.6 and 5 years, respectively. Furthermore, the variety of vegetables being consumed by the child increased slightly from consuming a mean of 2.1 different vegetables per day when the children were 1.6 years to 2.5 and 2.4 different vegetables per day when the children were 3.6 and 5 years, respectively.

### 3.1 Changes in Feeding Practices Over Time

Table 2 presents the means and standard deviations for each feeding practice at each time point, and Table 3 presents the regression coefficients that report the yearly change in feeding practices throughout the study. Of the 10 feeding practices, six were found to have significantly changed over time. Parents reported to use significantly more monitoring (*β* = 0.394, 95% CI 0.333–0.455, *p* < 0.001), food rewards (*β* = 0.256, 95% CI 0.217–0.294, *p* < 0.001), and restriction for health (*β* = 0.128, 95% CI 0.087–0.169, *p* < 0.001) per 1‐year increase in child age. The majority of the increase in use of monitoring, food rewards, and restriction for health occurred when the children were aged between 1.6 and 3.6 years, rather than between 3.6 years and 5 years. Changes in reported pressure to eat (*β* = 0.068, 95% CI 0.037–0.098, *p* < 0.001) and covert control (*β* = 0.043, 95% CI 0.014–0.071, *p* < 0.01) also reached statistical significance but with small increases per 1‐year increase in child age. Similarly, use of food for emotion regulation (*β* = −0.062, 95% CI −0.084 to −0.040, *p* < 0.001) showed a statistically significant change, with a small decrease per 1‐year increase in child age. The majority of the change in use of pressure and emotion regulation occurred when the children were aged between 1.6 and 3.6 years, rather than between 3.6 years and 5 years.

**Table 2:** Means and standard deviations of parent feeding practices at child age 1.6 years (T3), 3.6 years (T4), and 5 years (T5).

Feeding practice	T3	T4	T5
Pressure to eat	2.63 (0.87)	2.80 (0.92)	2.82 (0.94)
Food rewards	1.53 (0.67)	2.40 (1.02)	2.38 (1.05)
Modelling	4.44 (0.67)	4.45 (0.64)	4.47 (0.64)
Restriction for health	3.08 (0.95)	3.49 (0.98)	3.50 (1.00)
Monitoring	2.61 (1.63)	3.70 (1.18)	3.94 (1.06)
Encourage balance and variety	4.74 (0.32)	4.68 (0.38)	4.69 (0.36)
Emotion regulation	1.95 (0.57)	1.77 (0.56)	1.76 (0.59)
Availability	3.93 (0.26)	3.91 (0.32)	3.92 (0.30)
Covert control	3.20 (0.88)	3.26 (0.77)	3.31 (0.76)
Parent vegetable intake	2.73 (1.25)	2.76 (1.24)	2.73 (1.23)

*Note:* Feeding practice scores ranged between 1 and 5, with 5 indicating higher use of the feeding practice.

*Significant result *p* < 0.05, **Significant result *p* < 0.01, ***Significant result *p* < 0.001.

**Table 3:** Regression coefficients (β) and 95% confidence intervals (CI) are presented for each feeding practice representing the change in feeding practice score per one‐year increase in child age.

Feeding practice	*β* (95% CI)	*P*‐value
Pressure to eat	**0.068 (0.037; 0.098)**	**< 0.0001**
Food rewards	**0.256 (0.217; 0.294)**	**< 0.0001**
Modelling	0.016 (−0.008; 0.040)	0.195
Restriction for health	**0.128 (0.087; 0.169)**	**< 0.0001**
Monitoring	**0.394 (0.333; 0.455)**	**< 0.0001**
Encourage balance and variety	−0.013 (−0.027; 0.001)	0.073
Emotion regulation	**−0.062 (−0.084; −0.040)**	**< 0.0001**
Availability	0.003 (−0.009; 0.015)	0.627
Covert control	**0.043 (0.014; 0.071)**	**0.003**
Parent vegetable intake	−0.002 (−0.046; 0.043)	0.945

Significant findings are bolded.

### 3.2 Feeding Practice Association With Later Vegetable Consumption

Table 4 presents the regression coefficients for each feeding practice, representing the associated difference in vegetable consumption (quantity and variety) at T4 (child age = 3.6 years) and T5 (child age = 5 years) per unit difference in feeding practice. These models were adjusted for vegetable consumption at child age 1.6 years to predict the difference in associated vegetable consumption. Covert control and parent vegetable intake at T3 both significantly predicted increased vegetable quantity at T4. Every unit increase in covert control (β = 10.987, 95% CI 0.601–21.374, *p* < 0.05) and parent vegetable intake (*β* = 8.780, 95% CI 1.463–16.097, *p* < 0.05) at T3 was associated with the children consuming 11 g and 8.8 g more vegetables per day at T4 (2 years later). However, only parent vegetable intake (*β* = 12.225, 95% CI 2.647–21.803, *p* < 0.05) remained statistically significant at T5 (3.5 years later).

**Table 4:** Regression coefficients for each parental feeding practice association with later vegetable consumption representing change in vegetable consumption per day in response to one unit increase in use of feeding practice.

	Quantity β (grams/day)		Variety β (number of vegetables/day)	
T3 Feeding practice	T4	T5	T4	T5
Pressure to eat	−5.929	−0.105	−0.040	0.093
Food rewards	0.600	−11.740	−0.044	−0.211
Modelling	11.145	18.327†	0.186	0.247
Restriction for health	−5.985	3.424	−0.057	0.034
Monitoring	−4.660	−7.405†	−0.045	−0.123
Encourage balance & variety	21.039	3.167	−0.214	−0.156
Emotion regulation	−8.730	**−33.653** **	−0.016	−0.145
Availability	17.400	15.004	−0.032	0.368
Covert control	**10.987** *	11.220	0.201	0.181
Parent vegetable intake	**8.780** *	**12.225** *	0.021	0.145

*Note:* Bold values highlight significant results.

^†^ *p* = 0.051.

* Significant result *p* < 0.05.

** Significant result *p* < 0.01.

The other parental feeding practices showed significant prediction of vegetable quantity when the children were 5 years was use of food for emotion regulation. Every unit higher reported use of emotion regulation at child age 1.6 years was associated with children consuming 33.7 less grams of vegetables per day (*β* = −33.653, 95% CI −54.841 to −12.465, *p* < 0.01).

The sensitivity analysis was conducted without the covariate child vegetable consumption at T3; the direction of associations remained the same, but many of the effect sizes and significance levels increased (including for modelling, monitoring, and encourage balance and variety), suggesting baseline vegetable consumption was important in predicting later vegetable consumption. The sensitivity analysis findings are presented in a table in the Supporting Information.

### 3.3 Concurrent Changes in Parental Feeding Practices and Vegetable Consumption

Table 5 presents the regression coefficients for the six feeding practices which significantly changed over time. The coefficients represent predicted vegetable consumption change for every unit change in reported feeding practice per 1‐year increase in child age. While use of food rewards at T3 was not associated with later vegetable consumption, parents who began to use more food rewards throughout the study had children who consumed a lower quantity of vegetables per day on average by T5 (*β* = −11.633, 95% CI −17.717 to −5.549, *p* < 0.001). Furthermore, parents who began to use more covert control over time had children who consumed greater vegetable quantity by T5 (*β* = 13.555, 95% CI 6.374–20.737, *p* < 0.001) and a greater variety of vegetables per day (*β* = 0.209, 95% CI 0.064–0.354, *p* < 0.01).

**Table 5:** Regression coefficients for the concurrent changes in parental feeding practices and its subsequent association with vegetable consumption representing change in vegetable consumption per day in response to one unit increase in use of feeding practice.

Feeding practice	Vegetable quantity β (grams/day)	Vegetable variety β (number of vegetables/day)
Pressure	−5.127	−0.051
Food rewards	**−11.633** ***	−0.043
Restriction for health	−1.899	−0.009
Monitoring	0.013	0.034
Emotion regulation	−4.499	−0.132
Covert control	**13.555** ***	**0.209** **

*Note:* Bold values highlight significant results.

** Significant result *p* < 0.01,

*** Significant result *p* < 0.001.

## 4 Discussion

This study is the first to examine whether parental feeding practices changed across early childhood and whether any of these feeding practices (or changes in feeding practices) were associated with later vegetable consumption in children. Parents in the study reported greater use of many feeding practices as their children got older (namely between the ages of 1.6 and 3.6 years), in particular monitoring their children's food consumption, offering food as a reward, and restricting what their children eat. The study also found that greater use of covert control and parental vegetable consumption were associated with greater later vegetable consumption in young children. In contrast, using food to help with emotional regulation of their children and increasing use of food rewards over time were associated with lower later child vegetable consumption.

Parents reported to use five parental feeding practices more frequently as their children got older. Specifically, monitoring, food rewards, and restriction for health meaningfully increased; pressure and covert control slightly increased; and the use of food for emotion regulation slightly decreased over time. The limited previous research exploring the stability of feeding practices potentially suggests that parental feeding practices are relatively stable but can have small temporal changes, particularly in the earlier childhood years (Boots et al. 2018; Eichler et al. 2019; Taylor and Emmett 2018). Eichler et al. (2019) also reported slight increases in the use of food rewards and pressure to eat in children from 2 to 5 years of age, but in contrast to our findings, they found that the use of monitoring and restriction for health decreased over time. The findings between these studies may differ due to the younger age of first measurement in this study, or because different measurement tools for feeding practices were used. In the current study, the first measurement of feeding practices was done when the children were 1.6 years, but the study by Eichler et al. (2019) was done when children were 2 years. In this study, the biggest differences between time points were found between child ages 1.6 years and 3.6 years. Therefore, the earlier time point could be picking up the increase in food fussiness as the children are becoming more fussy at this age range (Taylor and Emmett 2018).

This study also points to parent vegetable consumption as being an important predictor of child vegetable consumption. Every one additional serve of vegetables a parent ate at child age 1.6 years was associated with their children eating between 8 and 12 grams more vegetables per day on average both 2 and 3.5 years later, respectively. Previous evidence suggests that parents who consume more vegetables have children who consume more vegetables, which supports the findings from this study (Ong et al. 2016). Since data was not collected on shared meal frequency, it was not possible to determine whether the effect of parental vegetable consumption on child vegetable consumption is acting through modelling or by another mechanism. Nonetheless, the CFPQ measure of modelling also demonstrated an effect of potential clinical relevance, because a unit increase in parental modelling predicted an 18.3 grams per day average increase in vegetable consumption 3.5 years later. However, this effect did not reach statistical significance (*p* = 0.051). Neither parent's vegetable intake nor modelling significantly changed over time.

Covert control reflects the parent controlling what the child can/cannot eat without the child being aware of the presence of that control, such as limiting the amount of unhealthy foods in the house. In this study, covert control was associated with higher subsequent child vegetable consumption, building on previous findings (Jarman et al. 2015). Every one standard deviation increase in covert control when children were 1.6 years was associated with children consuming 11 grams more vegetables per day 2 years later. Parents also reported to use slightly more covert control as their children got older. This is important as if parents were to start using more covert control throughout the period of the study, it was predicted that their children would consume 13 grams more vegetables per day on average per unit increase in report covert control. Given that children aged 2–3 years are recommended to consume 2.5 serves of vegetables per day (where one serve of vegetables is 75 g), this constitutes an increase of 17% of a serve. Parents could introduce covert control at any stage in early childhood and still potentially see improvements in their children's vegetable consumption. Therefore, it is never too late for parents to start developing a healthy home environment, such as using more covert control, modelling eating more healthy foods and eating more vegetables themselves.

Use of food for emotion regulation was not high on average and showed a small decrease over time, yet higher use was associated with substantially lower child vegetable consumption at 5 years. This could suggest that the practice is a problem for certain families, or is particularly problematic when introduced at 1.6 years of age. Use of food for emotion regulation has been linked to emotional eating and food fussiness (Tan and Holub 2015), which is associated with poorer diets and poor fruit and vegetable consumption (Santos et al. 2022). This is perhaps because when parents are giving their children food to regulate their emotions, the child may associate this emotion state with a more palatable food (typically high in fat and/or sugar), then displacing their vegetable intake (Santos et al. 2022). Parents in this study did report using slightly less emotion regulation as their children got older, but change in emotion regulation was not significantly associated with vegetable consumption. This would suggest that it is more important for parents to be mindful to limit their use of food to emotionally regulate their children right from the start of feeding.

While parents' use of food rewards at child age 1.6 years did not predict later vegetable consumption when children were aged 3.6 or 5 years, parents who reported increasing food rewards throughout the study had children who consumed less vegetables at 5 years, adding to previous findings (Edelson et al. 2016; Holley et al. 2016; Pullar et al. 2025). Additionally, food rewards were one of the feeding practices that parents in the study reported to use significantly more of as their children got older, which is particularly concerning due to the negative association with vegetable consumption suggested in this study. Interestingly, parents reported relatively low use of food rewards when children were 1.6 years. Therefore, future research should investigate the likely bi‐directional influences between child eating and parent use of rewards, and health promoters should look for ways to support parents to maintain low use of food rewards in the face of the challenges when children get older.

Of all the parental feeding practices (and concurrent changes in feeding practices), only the change over time in use of covert control was significantly associated with vegetable variety. While many feeding practices (e.g. modelling, covert control) may promote increased quantity of vegetable consumption, these findings indicate that covert control (most likely in terms of creating a healthy food home environment) may be beneficial to get children to eat a broader range of vegetables. It is also important to note that child consumption of vegetables at 1.6 years was controlled for in the regression models, to understand what associations and changes are present in vegetable consumption in relation to parental feeding practices from that point onwards. While statistically rigorous, it is possible that this approach offers conservative estimates of the impacts and importance of feeding practices, by adjusting for those differences in intakes already established in the first year of complementary feeding. The sensitivity analysis supports this hypothesis, with many effect sizes and significance levels increasing. Therefore, further research could explore the prospective associations between parental feeding practices and vegetable variety.

This paper is the first that uses longitudinal data on parental feeding practices over three time points (from ages 1.6 years to 5 years) and assesses the associations with child vegetable consumption. Building on limitations identified in a previous review (Pullar et al. 2025), this paper assessed multiple parental feeding practices over multiple time points and over a longer period than most previous research. This is beneficial as using longer follow‐ups and more time points allows for a better understanding of the temporal order and potential causal relationship between parental feeding practices and vegetable consumption. However, from this study, it is not possible to determine *why* parents may be changing their feeding practices. Previous research suggests that this is often in response to child behaviour (i.e., a child's temperament or fussiness), with bidirectional associations (Burnett et al. 2022) (Burnett et al. 2022). Secondly, a strength of this paper is that both vegetable quantity and variety were assessed, which is important as children need to consume a variety of vegetables to achieve a healthy diet (Conrad et al. 2018). Lastly, another strength was that the assessments of the children's diets were of high quality, done over multiple days.

However, this study has some limitations. Firstly, a significant proportion of participants from the original sample (48%) were not retained for this analysis, which leaves potential for attrition bias. However, this was explored, and the retained and full samples were comparable for all characteristics. Nonetheless, the retained sample of parents was highly educated with 61% of parents being educated to at least university level, which is somewhat higher than the population average of 52%–53% of women aged 25–44 years with tertiary education (Australian Bureau of Statistics 2026), potentially limiting generalisability to the wider parent population. Also, the majority of the sample were mothers, with one father completing the study at the follow‐up time points. Therefore, the findings from this study primarily reflect maternal feeding practices.

Additionally, without feeding practice data before children were aged 1.6 years, or child temperament measurement, this study cannot account for the potential interactions of these influences. Therefore, further work is needed to determine how child characteristics such as temperament may contribute to the developing relationship across time. Another limitation is that the InFANT dataset used for this analysis was collected from 2010 to 2014. Whilst there has been a time gap between data collection and analysis reported in this study, vegetable consumption in young children has remained relatively stable for the past 15 years (Health Survey for England 2020) and therefore these findings still bear relevance. Furthermore, this dataset remains one of the only known longitudinal datasets measuring multiple early childhood time points of rigorous dietary data collection in combination with parental feeding practices.

Lastly, the CFPQ and related items were used to measure parental feeding practices, which is a self‐report measure often done away from a mealtime. Therefore, it may not be a precise measure of parental feeding practices as it relies on how parents remember their mealtimes and does not differentiate between contexts (e.g., dinner vs. snacks). The Cronbach's alpha coefficients for the CFPQ, which evaluate how consistently the items measure the same construct, varied across time points/child ages, highlighting the complexity of measuring feeding practices. Cronbach's alpha coefficients did not always reach acceptable levels of reliability for ‘encouraging balance and variety’ and ‘restriction for health’. Removing the item ‘If I did not guide or regulate my child's eating, he/she would eat too many junk foods’ kept the Cronbach alpha scores for restriction for health more stable across the time points.

### 4.1 Conclusion

This longitudinal study has strengthened the evidence base examining the relationship between parent feeding practices and early childhood diets. The present study found that parents reported using monitoring, food rewards, and restriction for health significantly more often as their children aged from 1.6 to 5 years of age. Furthermore, higher use of structure‐based parental feeding practices such as parent vegetable intake and covert control predicted increases in children's vegetable consumption over time, while coercive feeding practices such as use of food for emotion regulation and increasing food rewards predicted reduced vegetable consumption in young children at 5 years. Future research should continue to explore the longitudinal relationships between parental feeding practices and vegetable consumption, but specifically in terms of why feeding practices may change over time and explore further the influences on vegetable variety. Additionally, interventions and practice should focus on supporting parents to model eating vegetables, implement covert control, and prevent increases in the offering of food rewards over time.

## Author Contributions

K.J.C. co‐designed and co‐led the Melbourne INFANT Programme trial and follow‐up. A.C.S. contributed to data collection. L.P. designed the analysis plan, conducted the analysis, interpreted the results, drafted the manuscript, and undertook revisions. A.C.S., M.J., J.B., K.J.C. and A.B. contributed to the study design and interpretation of results. All authors contributed to revision of the manuscript, and all read and approved the final manuscript.

## Conflicts of Interest

The authors declare no conflicts of interest.

## Supporting information


Supporting File 1

Supporting File 2

Supporting File 3

## Data Availability

The data that support the findings of this study are available from the corresponding author upon reasonable request.
