# Supplementary material for: Exploring Changes in Parental Feeding Practices With Young Children Over Time and Their Influence on Young Children's Vegetable Consumption
Source: Matern Child Nutr. 2026 Sep 14;22(3):e70238. doi: 10.1111/mcn.70238 (PMC13572970; doi:10.1111/mcn.70238)
Supplement: Supplementary file 1 [file MCN-22-e70238-s001.docx]

| **T1 Characteristics** | | **Included Sample (n = 255)** | **Base Sample (n = 530)** |
| --- | --- | --- | --- |
| **Parent** | ***Age (Years)*** | 32.79 (4.12) | 32.3 (4.31) |
|  | ***Relation to child*** |  |  |
|  | Mother | 255 (100%) | 529 (99.8%) |
|  | ***Education*** |  |  |
|  | Trade / Diploma | 100 (39.22%) | 243 (45.85%) |
|  | University or higher | 155 (60.78%) | 287 (54.15%) |
|  | ***BMI*** | 24.01 (4.72) | 24.51 (5.29) |
|  | ***Living with partner*** |  |  |
|  | Yes | 252 (98.82%) | 521 (98.3%) |
|  | | | |
| **Child** | ***Age (Years)*** | 0.31 (0.12) | 0.33 (0.13) |
|  | ***Sex*** |  |  |
|  | Boy | 135 (52.94%) | 285 (52.58%) |
|  | Girl | 120 (47.06%) | 257 (47.42%) |
|  | ***zBMI*** | -0.46 (1.04) | -0.46 (1.04) |
|  | ***Group*** |  |  |
|  | Intervention | 129 (50.59%) | 271 (50.0%) |
|  | Control | 126 (49.41%) | 271 (50.0%) |

**Descriptive characteristics at T1 for the included sample and base samples from the InFANT project.**
