# Supplementary material for: Exploring Changes in Parental Feeding Practices With Young Children Over Time and Their Influence on Young Children's Vegetable Consumption
Source: Matern Child Nutr. 2026 Sep 14;22(3):e70238. doi: 10.1111/mcn.70238 (PMC13572970; doi:10.1111/mcn.70238)
Supplement: Supplementary file 2 [file MCN-22-e70238-s002.docx]

| **Feeding practice** | **Number of items** | **T3 Alpha** | **T4 Alpha** | **T5 Alpha** |
| --- | --- | --- | --- | --- |
| Pressure to Eat CFPQ | 4 | 0.694 | 0.703 | 0.733 |
| Food Rewards CFPQ | 3 | 0.606 | 0.711 | 0.754 |
| Modelling CFPQ | 4 | 0.805 | 0.762 | 0.799 |
| Restriction for health CFPQ | 3 | 0.475 | 0.614 | 0.731 |
| Monitoring CFPQ | 4 | 0.753 | 0.631 | 0.652 |
| Encourage Balance & Variety CFPQ | 4 | 0.350 | 0.532 | 0.602 |
| Emotion Regulation CFPQ | 3 | 0.657 | 0.658 | 0.736 |
| Availability | 1 | N/A | N/A | N/A |
| Parent Vegetable Intake | 1 | N/A | N/A | N/A |
| Covert Control | 5 | 0.788 | 0.790 | 0.819 |

A table to show the internal consistency of each construct from its measurement. A score of 0.6 or more indicates a moderate level of agreement.

*We excluded one item from ‘restriction for health’ feeding practice as the alpha with all four items was so low at T5 (α = 0.164). Removing this item ensured more stability at all time points for this variable.*
