# Supplementary material for: Exploring Changes in Parental Feeding Practices With Young Children Over Time and Their Influence on Young Children's Vegetable Consumption
Source: Matern Child Nutr. 2026 Sep 14;22(3):e70238. doi: 10.1111/mcn.70238 (PMC13572970; doi:10.1111/mcn.70238)
Supplement: Supplementary file 3 [file MCN-22-e70238-s003.docx]

Regression coefficients for each parental feeding practice association with later vegetable consumption representing change in vegetable consumption per day in response to one unit increase in use of feeding practice. This table shows coefficients from sensitivity analysis that was done without the covariate child vegetable consumption at T3.

| **Sensitivity Analysis** | **Quantity β (grams/day)** | | **Variety β (number of vegetables/day)** | |
| --- | --- | --- | --- | --- |
| **T3 Feeding practice** | **T4** | **T5** | **T4** | **T5** |
| Pressure to Eat | -5.64 | -1.82 | -0.117 | 0.027 |
| Food Rewards | -1.99 | -14.90 | -0.127 | -0.275^†^ |
| Modelling | **19.45**** | **21.70*** | 0.296^†^ | 0.310^†^ |
| Restriction for health | -5.88 | 2.08 | -0.019 | 0.042 |
| Monitoring | **-6.26*** | **-7.95*** | -0.077 | **-0.148*** |
| Encourage Balance and Variety | **28.93*** | 3.48 | -0.078 | -0.109 |
| Emotion Regulation | -7.91 | **-31.33**** | -0.102 | -0.223 |
| Availability | 21.99 | 19.18 | 0.166 | 0.524 |
| Covert Control | **14.35**** | 13.11^†^ | 0.189 | 0.176 |
| Parent Vegetable Intake | **12.25***** | **13.89**** | 0.147^†^ | **0.201*** |

*Significant findings are bolded.* *Highlighted findings were findings that were not statistically significant in the included analysis but were statistically significant in the sensitivity analysis. † p = 0.051, *Significant result p < .05, **Significant result p < .01, ***Significant result p < .001.*
